# Neurological manifestations in patients with symptomatic COVID-19 admitted to the Bafoussam Regional Hospital, Cameroon

**DOI:** 10.11604/pamj.2021.38.326.28973

**Published:** 2021-04-05

**Authors:** Yannick Fogoum Fogang, Michel Noubom, Pierre-Yves Bassong, Paul Chimi Mbonda, Iliassou Njoudap Mfopou, Daniel Massi Gams, Callixte Tegueu Kuate, Joseph Kamtchum-Tatuene

**Affiliations:** 1 Faculty of Medicine and Pharmaceutical Sciences, University of Dschang, Dschang, Cameroon; 2 Bafoussam Regional Hospital, Bafoussam, Cameroon,; 3 Faculty of Medicine and Pharmaceutical Sciences, University of Bamenda, Bamenda, Cameroon,; 4 Faculty of Medicine and Biomedical Sciences, University of Yaounde I, Yaounde, Cameroon,; 5 Faculty of Health Sciences, University of Buea, Buea, Cameroon,; 6 Neuroscience and Mental Health Institute, Faculty of Medicine and Dentistry, University of Alberta, Alberta, Canada

**Keywords:** Coronavirus, headache, anosmia, COVID-19, Africa

## Abstract

**Introduction:**

although the main manifestations of COVID-19 are respiratory, several neurological symptoms and complications have also been reported. The pandemic seems to have some epidemiological specificities in sub-Saharan Africa, and this may be reflected in the type and frequency of neurological symptoms. This study aimed to report neurological manifestations associated with symptomatic COVID-19 in a sub-Saharan African setting.

**Methods:**

we conducted a retrospective review of symptomatic PCR-confirmed COVID-19 cases admitted to the Bafoussam Regional Hospital between March and September 2020. Patients’ files were reviewed at discharge by a consultant neurologist. Socio-demographic characteristics, co-morbidities, symptoms on admission, neurological symptoms during hospitalization, management, and in-hospital outcome were recorded. Comparisons between patients with and without neurological symptoms were performed using Fisher’s exact and Mann-Whitney U test.

**Results:**

we enrolled 177 symptomatic patients (68% men). Mean age was 54.6 ± 17.8 years (range 2-99 years). Co-morbidities were present in 57.6% of patients, including hypertension (27.1%) and diabetes mellitus (25.4%). Neurological symptoms were found in 113 (63.8%) patients. The most frequent were headache (39.0%), myalgia (35.6%), anosmia (11.9%), impaired consciousness (10.7%) and delirium (5.6%). Regarding the presenting symptoms, fever was more frequent in patients with neurological symptoms than in those without (81.4% versus 50.0%, p< 0.001), while digestive symptoms were less frequent in patients with neurological symptoms (0.9% versus 9.4%, p= 0.004).

**Conclusion:**

neurological manifestations are frequent and heterogeneous in patients with symptomatic COVID-19. Further studies are needed to clarify the pathophysiology of neurological symptoms in COVID-19 and their impact on patients’ long-term outcome.

## Introduction

The COVID-19 pandemic, caused by the Severe Acute Respiratory Syndrome Coronavirus 2 (SARS-CoV-2), represents a tremendous challenge for healthcare systems, healthcare workers and the general population all over the globe. Although Africa is considered by many as relatively spared by the pandemic, the latter has caused important damages to the continent characterized by low financial resources and fragile health systems [1]. Cameroon was among the first African countries to report cases of COVID-19 [2]. The three main cities of the country, namely Yaoundé, Douala and Bafoussam are the most affected [2, 3]. To date, Cameroon has officially reported 38988 positive cases with 588 deaths [4]. As in many other countries, these figures are underestimated due to the suboptimal availability of mass testing [1]. Although the manifestations of COVID-19 are predominantly respiratory, neurological complications have also been reported, involving both the peripheral and the central nervous systems with early alteration of olfaction and taste due to the tropism of the SARS-CoV-2 for the olfactory mucosa [5-11].

Rare cases of patients with myopathies have also been reported although formal causal relationship with infection by SARS-CoV-2 was not established [9]. The prevalence of neurological manifestations in COVID-19 ranges from 3.5% to 84% across studies, thus suggesting the contribution of sampling bias [6, 9]. The neurological manifestations are thought to result from para-infectious or post-infectious immune-mediated mechanisms, or toxic-metabolic effects of severe pulmonary or systemic SARS-CoV-2 infection [7, 9]. To date, there is no definitive evidence supporting the contribution of direct neuronal infection to the development of encephalopathy, delirium, and other neurological manifestations observed in patients with COVID-19 [12]. In Northern Africa, neurological manifestations of COVID-19 have been reported in several hospital-based cross-sectional studies [13-15]. On the contrary, apart from a few case reports [16-18], data on the neurological manifestations of COVID-19 in sub-Saharan Africa are scarce. This study aimed to determine the frequency and types of neurological symptoms in patients with COVID-19 in Bafoussam, a semi-urban sub-Saharan setting.

## Methods

**Study setting:** the Bafoussam Regional Hospital is a tertiary referral hospital for the West region of Cameroon with a capacity of 235 beds. It was chosen by health authorities for the management of symptomatic COVID-19 patients. This hospital receives symptomatic patients with suspected or confirmed COVID-19 infection for better management. HIV infection and malaria are endemic in Cameroon. In the West region, the population prevalence of HIV infection is 1.8%, and the incidence of malaria is 70 per 1000 individuals per year [19].

**Study design and participants:** we conducted a retrospective chart review of symptomatic PCR-confirmed COVID-19 cases (nasopharynx swab) admitted to the Bafoussam Regional Hospital between March and September 2020. All patients were treated by a multidisciplinary team according to national health authorities´ recommendations. This treatment includes antibiotics (azythromicin, amoxicillin and clavulanic acid), hydroxychloroquin, anticoagulants, dexamethasone, antipyretics, and treatment of comorbidities. Therapeutic doses of anticoagulants are administered for severe cases, and prophylactic doses for moderate cases. For mild cases, the treatment is administered orally. The following biological tests were performed for all patients: full blood count, C-reactive protein, blood urea nitrogen and creatinine, blood sodium, chloride and potassium levels, liver enzymes SGPT/SGOT, blood sugar, HIV test (Determine test®), rapid test for malaria (Caretest Malaria HRP2/pLDH, (Pf/PAN) combo®), and D-Dimers. Brain imaging, cerebrospinal fluid analysis, and other biological tests were requested when deemed necessary.

**Data collection:** a consultant neurologist (YF) reviewed patients´ chart at discharge and recorded socio-demographic characteristics (age and gender), co-morbidities (hypertension, diabetes mellitus, smoking, alcohol consumption, chronic heart failure, chronic kidney disease, human immunodeficiency virus (HIV) infection, obesity, chronic bronchitis, sickle cell disease, malignancy, hepatitis B and C viral infection, epilepsy and schizophrenia), symptoms on admission (cough, fever, dyspnea, fatigue, myalgia, headache, impaired consciousness, delirium, hemiplegia, rhinorrhea, anosmia, ageusia, sore throat, chest pain, digestive and other symptoms), neurological symptoms during hospitalization (headache, myalgia, anosmia, impaired consciousness, delirium, panic attack, hemiplegia, ageusia, aphasia, dysphagia, neck stiffness, hypersialorrhea, ataxia, refractory hiccups, dysphonia, tinnitus), management (antibiotics, hydroxychloroquin, anticoagulants and oxygen therapy), and in-hospital outcome (death or alive). Disease severity was established according to the World Health Organization (WHO) classification [20].

**Data analysis:** data were summarized as frequency with percentage for categorical variables and median with interquartile range for continuous variables, unless otherwise stated. Comparisons between patients with and without neurological symptoms were made using Fisher´s exact test for categorical variables and Mann-Whitney U test for continuous variables. Statistical significance was defined as p ≤0.05. Statistical analyses were performed with STATA (version 16, StataCorp, College Station, TX, USA).

**Ethics approval:** the study was approved by the Regional Ethics Committee and was conducted in accordance with the 1964 Helsinki Declaration and its subsequent amendments. Due to the emergency context of COVID-19 pandemic, the requirement for patients´ written informed consent was waived.

## Results

From March to September 2020, 241 suspected cases of symptomatic COVID-19 were identified at the Bafoussam Regional Hospital COVID-19 unit, including 177 RT-PCR confirmed symptomatic (68% men) and 64 non confirmed cases.

**Demographic and clinical characteristics:** the mean age of symptomatic patients was 54.6 ± 17.8 years (range 2-99 years). Comorbidities were recorded in 57.6% of the study population. The most frequent where hypertension (27.1%) and type 2 diabetes mellitus (25.4%) (Table 1). The prevalence of HIV infection and malaria was 3.4% and 2.8%, respectively. All cases of malaria were found in patients with neurological symptoms. Cases of COVID-19 were classified according to the World Health Organization as mild, moderate, or severe in 25.3%, 34.9% and 39.8% of patients respectively, with no significant difference between the groups of patients with and without neurological symptoms (Table 1).

**Table 1:** socio-demographic characteristics and co-morbidities of patients with symptomatic COVID-19 according to the presence of neurological symptoms*

		Total (n = 177)	Neurological symptoms		p-value
			Yes (n = 113)	No (n = 64)	
Age, mean± SD		54.6±17.8	55.8±16.6	52.5±19.5	0.22
Men, n (%)		120 (67.8)	78 (69.0)	42 (65.6)	0.74
Severity** (WHO classification)	Mild, n (%)	42 (25.3)	23 (20.5)	19 (35.2)	0.12
	Moderate, n (%)	58 (34.9)	43 (38.4)	15 (27.8)	
	Severe, n (%)	66 (39.8)	46 (41.1)	20 (37.0)	
Hypertension		48 (27.1)	26 (23.0)	22 (34.4)	0.12
Diabetes mellitus		45 (25.4)	28 (24.7)	17 (26.6)	0.86
Smoking		13 (7.3)	10 (8.9)	3 (4.7)	0.38
Alcohol consumption		12 (6.8)	9 (8.0)	3 (04.7)	0.54
Chronic heart failure		8 (4.5)	4 (3.5)	4 (6.3)	0.46
Chronic Kidney disease		6 (3.4)	2 (1.8)	4 (6.3)	0.19
HIV infection		6 (3.4)	4 (3.5)	2 (3.13)	0.99
Malaria		5 (2.8)	5 (4.4)	0 (0.0)	0.006
Obesity		3 (1.7)	2 (1.8)	1 (1. 6)	0.99
Chronic bronchitis		3 (1.7)	1 (0.9)	2 (3.1)	0.91
Stroke		2 (1.7)	2 (1.8)	0 (0.0)	0.54
Chronic headache		1 (0.6)	1 (0.9)	0 (0.0)	0.99
Sickle cell disease		1 (0.6)	0 (0.0)	1 (3.13)	0.36
Malignancy		2 (1.1)	0 (0.0)	2 (3.1)	0.13
HCV infection		1 (0.6)	0 (0.0)	1 (1.6)	0.99
HBV infection		1 (0.6)	0 (0.0)	1 (1.6)	0.36
Epilepsy		1 (0.6)	1 (0.9)	0 (0.0)	0.99
Schizophrenia		1 (0.6)	0 (0.0)	1 (1. 6)	0.99
No comorbidity		75 (42.4)	52 (46.0)	23 (35.9)	0.21

* In the entire table, categorical variables are summarized as count (proportion in the corresponding group). ** Information available for 166 patients only (112 with neurological symptoms and 54 without)

**Symptoms on admission:** respiratory symptoms and fever where the most frequent presenting complaints. Cough, dyspnea, and fever were reported by 73.5%, 70.1% and 48.0% of patients, respectively (Table 2). Fever was more frequent in patients with neurological symptoms than in those without (81.4% versus 50.0%, p <0.001). Fever was present in 58.7% of patients with headache and 53.3% of patients with myalgia. Digestive symptoms (abdominal pain, diarrhea, vomiting, gastro-intestinal tract bleeding) were more frequent in patients without neurological symptoms (Table 2). Two patients presented with hemiplegia and were further diagnosed with stroke.

**Table 2:** presenting complaints in patients with symptomatic COVID-19 according to the presence of neurological symptoms

Presenting Symptoms*	Study population n (%)	Neurological symptoms**		p-value
		Yes (n=113)	No (n=64)	
Cough	130 (73.5)	85 (75.2)	45 (70.3)	0.48
Fever	124 (70.1)	92 (81.4)	32 (50)	<0.001
Dyspnea	85 (48.0)	59 (52.2)	26 (40.6)	0.14
Fatigue	62 (35.0)	44 (38.9)	18 (28.1)	0.15
Myalgia	51 (28.8)	51 (45.1)	-	
Headache	49 (27.7)	49 (43.4)	-	
Impaired consciousness	3 (1.7)	3 (2.7)	-	
Delirium	7 (4.0)	7 (6.2)	-	
Hemiplegia	2 (1.1)	2 (1.8)	-	
Rhinorrhea	12 (6.8)	10 (8.9)	2 (3.1)	0.15
Anosmia	8 (4.5)	8 (7.1)	-	
Sore throat	6 (3.4)	4 (3.5)	2 (3.1)	0.88
Chest pain	5 (2.8)	2 (1.8)	3 (4.7)	0.26
Digestive*** symptoms	7 (4.0)	1 (0.9)	6 (9.4)	0.004
Others****	7 (4.0)	5 (4.4)	2 (3.1)	0.93

* In the entire table, data are presented as count (proportion in the corresponding group). **Empty cases correspond to neurological symptoms which logically, are not present in patients with no neurological symptoms. ***Digestive symptoms include diarrhea, vomiting, abdominal pain and gastro-intestinal tract bleeding. ****Others include bleeding, hypersialorrhea, pelvic pain, swollen and painful leg (in a patient with deep venous thrombosis), and conjunctivitis.

**Neurological symptoms and outcome:** neurological symptoms were found in 113 (63.8%) patients (Table 3). The most frequent were headache (39.0%), myalgia (35.6%), and anosmia (11.9%). There were 5 cases of hemiplegia due to stroke, including three that occurred during hospitalization. During hospitalization, three patients had a stroke, sixteen patients developed an impairment of consciousness and three patients had a delirium. For these patients, the median time to occurrence of neurological symptoms after admission was 4 days (range: 1 to 20 days). The median time from symptoms onset to admission was 7 days (IQR: 5-14), and the median length of hospitalization was 14 days (IQR: 9-19). There was no significant difference between patients with and without neurological symptoms regarding the delay from symptoms onset to admission and the length of stay (Table 4).

**Table 3:** frequency of neurological symptoms in patients with symptomatic COVID-19

Neurological symptoms*	study population (n =177)
Headache, n (%)	69 (39.0)
Myalgia	63 (35.6)
Anosmia	21 (11.9)
Impaired consciousness	19 (10.7)
Delirium	10 (5.6)
Panic attack	5 (2.8)
Hemiplegia/-paresia	5 (2.8)
Ageusia	5 (2.8)
Aphasia	3 (1.7)
Dysphagia	2 (1.1)
Neck stiffness	2 (1.1)
Hypersialorrhea	2 (1.1)
Ataxia	1 (0.6)
Refractory hiccups	1 (0.6)
Dysphonia	1 (0.6)
Tinnitus	1 (0.6)

* In this table, we present neurological symptoms present on admission or identified during hospitalization.

**Table 4:** management and outcome of patients with symptomatic COVID-19

	Total	Neurological symptoms		p-value
		Yes (n=113)	No (n=64)	
Delay from symptoms onset to admission (days)*, median (IQR)	7 (5 - 14)	7 (5 - 10)	7 (5 - 14)	0.34
Oxygen therapy**, n (%)	71 (40.3)	51 (45.5)	20 (31.3)	0.08
Mortality, n (%)	53 (29.9)	35 (31.0)	18 (28.1)	0.41
Delay from admission to death (days)***, median (IQR)	2 (1 - 3)	2 (1 - 3)	2 (1 - 4)	0.78
Length of stay (days)****, median (IQR)	14 (9 - 19)	14 (9 - 19)	14 (13 - 19)	0.63

* Information available only for 167 patients (107 with neurological symptoms and 60 without). ** Information available for 176 patients (112 with neurologic symptoms and 64 without). *** Information available only for 52 patients (35 with neurological symptoms and 17 without).

Cerebrospinal fluid (CSF) analysis was performed in 11 patients and showed pleiocytosis in three cases, with 6, 13 and 42 cells/mm^3^ with a predominance of mononuclear cells. The CSF protein concentration ranged from 0.3 to 1.2 g/l. CSF glucose concentration was normal in all eleven cases. Brain computed tomography (CT) scan was obtained only in patients with a focal motor deficit (five cases) and showed a left middle cerebral artery ischemic stroke in two cases. In one case, we found multiple lacunar infarcts. In the remaining two cases, the brain CT scan was normal. Four patients diagnosed as stroke had at least one cardiovascular risk factor (hypertension, diabetes mellitus, or chronic heart failure).

Patients were treated with antibiotics, notably amoxicillin with clavulanic acid (94.9%), azithromycin (56.3%) and hydroxychloroquin (44.3%). Anticoagulants (enoxaparin) were prescribed to 60.8% of patients, and oxygen to 40.3% (Table 4). Mortality rate was 29.9%. There was no significant difference in mortality between the patients with neurological symptoms and those without (31.0% versus 28.1%, p=0.3) (Table 4). In fatal cases, the median duration of hospitalization was 2 days (IQR: 1-3). Patients with impaired consciousness (n=19 of 177) had a higher mortality rate than those without (84.2% versus 23.4%, p<0.001).

## Discussion

This is one of the first studies on neurological symptoms of COVID-19 in sub-Saharan Africa. We found that mean age of patients with COVID-19 and neurological symptoms was 55 years, and more than two thirds were male. Neurological symptoms were present in 63.8% of patients and were most frequently associated with fever, and less frequently with digestive symptoms. Headache, myalgia, anosmia, and impaired consciousness were the main neurological symptoms. Findings regarding age and sex distribution of patients are in line with previously published data on patients with COVID-19 and neurological symptoms [21-23]. Previous studies have reported a frequency of neurological symptoms ranging from 6% to 38% in patients with COVID-19 [21-24]. The high frequency of headaches in our study compared to that of preliminary studies conducted in China (39% versus 8% and 13% for Chachkhiani *et al*. and Mao *et al*. [22, 23]) may contribute to the overall difference.

Headaches were reported in 39% of the patients in our study. Initial reports in China found headache in 6-13% of patients with COVID-19 [23, 25-27]. A subsequent study found headache in 40% of patients [28]. Two studies in Europe reported headache in 23.7% and 74.6% of cases [29, 30], and in the latter study, headaches with migraine like-features were present in almost 25% of patients [30]. In those studies, headache was associated with fever, low mortality [29], anosmia, ageusia, and a shorter time to recovery [30]. The discrepancies in the frequency of headache between studies can be explained by the methodology used and the qualification of the investigators. Prospective studies targeting headache reported higher frequencies of headache and a better prognosis, probably because most patients included had mild forms of COVID-19 and could clearly report a headache contrary to patients with severe forms. Headache in the context of COVID-19 may be attributed to the activation of the trigemino-vascular system by circulating pro-inflammatory cytokines [30], associated or not with meningitis.

Myalgia was reported in 35.6% of patients. Our results are in line with those published previously, where myalgia was reported in 31% to 50% of patients with COVID-19 [23, 28, 31, 32]. Two studies found an increased creatine kinase level in 33% of patients [21, 33]. The putative underlying mechanism to explain myalgia in SARS-CoV-2 infection is the effect of systemic inflammatory cytokines on muscle fibers [21]. In China, initial reports found anosmia and ageusia in 5.1% and 5.6% respectively [23]. In two prospective European studies, 86% and 85.6% of patients with COVID-19 had an olfactive dysfunction, 86% and 88% had a gustatory dysfunction [34, 35]. The high frequency of olfactory and gustatory dysfunction in European studies can be explained by their study design, where taste and smell where systematically examined including subclinical deficits [34, 35] in patients with mild to moderate COVID-19. This was not the case in retrospective studies, where these symptoms were not systematically reported, especially in patients with severe forms of the disease.

Abnormal smell and taste are both strongly associated with COVID-19 [36], and are more frequently reported in COVID-19 patients than in a historical cohort of influenza patients [34]. These symptoms have been recommended by the American Academy of Otolaryngology-Head and Neck Surgery and the British Association of Otorhinolaryngology as part of primary screening symptoms for COVID-19 [5]. Accumulating evidence suggest that the neurotropism of SARS-CoV-2 results in the invasion of the olfactory nerve which extends into the olfactory cortex, basal ganglia, midbrain and brain stem [37-40]. Although the virus can enter the brain, it seems to predominantly infect vascular and immune cells rather than neurons [12]. Impaired consciousness and delirium were present in 16.3% of patients and associated with a high mortality. In a study from China, impaired consciousness was found in 14.8% of hospitalized patients with COVID-19, and was also significantly associated with increased mortality [23]. In the latter study, impaired consciousness was reported in 22% of fatal cases, versus 1% among patients who recovered.

Encephalopathy in patients with COVID-19 may be attributed to the effect of one or more of these factors on the brain: severe hypoxemia, inflammation (cytokine storm), acute cerebrovascular disease, direct viral invasion and non-convulsive status epilepticus. In a neuroimaging study of patients with COVID-19 and neurological manifestations, including 93% with altered mental status, 56% had brain MRI abnormalities, notably ischemic stroke (27%), leptomeningeal enhancement (17%) and encephalitis (13%) [41]. Another study analyzing cerebrospinal fluid (CSF) in four patients with COVID-19 related encephalopathy, found a significant increase in CSF inflammation biomarkers in contrast with a normal white blood cell count [42]. It is difficult to determine the exact cause of encephalopathy in all the cases due to a high early mortality, limited resources (health personnel, imaging and laboratory work-up) aggravated by an important flow of patients and the risk of viral transmission to health personnel.

In our study, five (2.1%) patients had a confirmed diagnosis of stroke. The frequency of stroke during COVID-19 varies from 1% to 6% [23, 43-45], with a predominance of ischemic stroke due to large artery disease. In the study by Kremer *et al*. stroke was reported in 27% of COVID-19 patients with neurological manifestations and was the most frequent neuroimaging finding. Patients with stroke were less often diagnosed with acute respiratory distress and among them, two-third had a large artery infarction and one third a watershed cerebral infarction [41]. The frequency of stroke in our study might have been higher if brain imaging was systematically performed in patients with neurological symptoms, given that stroke can present with isolated impaired consciousness or brainstem symptoms [46]. Four out of five patients who had a stroke had known vascular risk factors. Poor follow-up of patients with risk factors of stroke during the pandemic [47], added to SARS-CoV-2 infection could have increased the risk of stroke. Given the high prevalence of COVID-19 in the general population, it is also possible that our patients had a stroke while infected by SARS-CoV-2, without a causal relationship between the two pathological processes. In a case series, patients with stroke and COVID-19 had a similar epidemiological profile compared to stroke patients without COVID-19. They had significant stroke risk factors, and were generally older [48].

The pathophysiology of stroke in COVID-19 is attributed to a state of hypercoagulability, inflammation and endothelial dysfunction [49], that may increase the risk of stroke especially in those with a high burden of traditional cerebrovascular risk factors. We recorded other neurological symptoms like ataxia, dizziness, dysphagia, refractory hiccups and hypersialorrhea that were less frequent. These symptoms can be the manifestation of inflammatory, vascular, hypoxic and/or direct viral lesions involving brainstem, cerebellar and vestibular pathways [50]. A better availability of and accessibility to brain magnetic resonance imaging (MRI) would have helped us to better investigate these symptoms.

Our study has several limitations. First, obtaining a detailed neurological assessment and investigation is challenging in patients who are often critically ill and contagious, yet limiting the opportunity to delineate the underlying mechanism. Second, given that this study is hospital-based and retrospective in nature, it is more likely that it overestimates the prevalence of neurological symptoms in patients with COVID-19. Indeed, only symptomatic patients with COVID-19 referred for diagnostic confirmation and management were enrolled. Moreover, some non-specific neurological symptoms could be explained by concurrent HIV infection or malaria. However, the relatively low prevalence of these endemic infections in our sample suggests that their contribution to the reported neurologic symptoms is limited. Third, the relatively small size of our study population prevented us from carrying out some advanced analyses, including multivariable regression to identify predictors of poor outcome (death, prolonged hospitalization) in patients with and without neurological symptoms.

## Conclusion

Neurological symptoms are frequent, present in nearly two-thirds of patients with symptomatic COVID-19, and heterogenous in nature. Headache, myalgia, anosmia, impaired consciousness, and delirium were the most common symptoms in patients admitted to the Bafoussam Regional Hospital. In term of neurological specificity, anosmia was the main finding. Despite its limitations, this study provides important preliminary epidemiological data to guide the screening and management of neurological symptoms in patients with COVID-19. Whenever possible, prompt neurological assessment should be incorporated in the management of patients with COVID-19 to ensure adequate diagnosis and reporting of neurological complications, and to organize the long-term tracking of neurological sequelae. Further studies are needed to clarify the pathophysiology of neurological symptoms in COVID-19 and their impact on patients´ long-term outcome.

### What is known about this topic

- Neurological manifestations associated with COVID-19 are being reported;
- Central and peripheral nervous system, and muscles can be affected during COVID-19;
- Nervous system involvement can be attributed to the direct effects of the virus, para-infectious or post-infectious immune-mediated manifestations, or complications of the systemic effects of COVID-19.

### What this study adds

- This is one of the first studies providing detailed clinical data on neurological symptoms of COVID-19 in sub-Saharan Africa;
- Neurological symptoms are present in 63.8% of patients admitted with symptomatic COVID-19;
- Fever is more frequent, and digestive symptoms less frequent in patients with neurological symptoms than in those without.
